# The reporting of CONSORT items and key feasibility elements in pilot and feasibility studies in hip and knee arthroplasty is suboptimal: a methodological survey

**DOI:** 10.1186/s40814-026-01859-x

**Published:** 2026-06-04

**Authors:** Zinnia Chung, Leigh-Ann Grant, Lawrence Mbuagbaw, Lipalo Mokete, Mohit Bhandari, Lehana Thabane

**Affiliations:** 1 Michael G. DeGroote School of Medicine, McMaster University, Hamilton, ON Canada; 2 Department of Health Research Methods, Evidence and Impact, McMaster University, Hamilton, ON Canada; 3 Department of Anesthesia, McMaster University, Hamilton, ON Canada; 4 Department of Pediatrics, McMaster University, Hamilton, ON Canada; 5 Biostatistics Unit, Father Sean O’Sullivan Research Centre, St. Joseph’s Healthcare, Hamilton, ON Canada; 6 Centre for Development of Best Practices in Health (CDBPH), Yaoundé Central Hospital, Yaoundé, Cameroon; 7 Division of Epidemiology and Biostatistics, Department of Global Health, Stellenbosch University, Cape Town, South Africa; 8 Division of Orthopaedics, University of the Witwatersrand, Johannesburg, South Africa; 9 Department of Surgery, McMaster University, Hamilton, ON Canada; 10 Research Institute of St. Joeseph’s Hamilton, St Joseph’s Healthcare Hamilton, Hamilton, ON Canada; 11 Faculty of Health Sciences, University of Johannesburg, Johannesburg, South Africa

**Keywords:** Pilot and feasibility trials, Pilot trials, Feasibility studies, Hip arthroplasty, Knee arthroplasty, Hip replacement;, Knee replacement, Methodological survey

## Abstract

**Background:**

As the global demand for hip and knee arthroplasty procedures grow, advancements in orthopedic research are vital for progress. Pilot and feasibility trials play a critical role in optimising research efficiency, highlighting methodological gaps, and maximising study insights. However, the completeness of reporting in hip and knee arthroplasty pilot and feasibility trials remains unclear.

**Objectives:**

The primary objective was to evaluate reporting completeness, assessed using the CONSORT checklist extension to pilot and feasibility trials. Secondary objectives included evaluating the reporting of key feasibility items and exploring factors associated with reporting completeness.

**Setting and methods:**

This methodological survey analysed pilot and feasibility trials in hip and knee arthroplasty. A PubMed search identified relevant manuscripts published between January 1, 2017, and December 31, 2023. A minimum acceptable sample size of 147 (of 278 eligible) was identified, based on a calculation involving an estimated reporting completeness of 25% of the CONSORT checklist, a 95% confidence interval, and a 5% margin of error. Descriptive statistics were reported, and a multivariable linear regression with robust (HC1) standard errors was completed.

**Results:**

Of 278 eligible publications identified from PubMed, a random sample of 147 studies was included. Reporting completeness was low, with studies accounting for a mean of 54.98% (16.95) of applicable CONSORT extension items. Notably, pilot-specific objectives and future study implications were often underreported. Similarly, manuscripts missed approximately 71.35% (10/14) of key feasibility elements. Studies that referenced reporting guidelines in the manuscript text were associated with better reporting, while those that failed to disclose their funding sources and lacked more key feasibility items demonstrated weaker reporting completeness.

**Conclusion:**

Hip and knee arthroplasty pilot and feasibility trials exhibit suboptimal reporting completeness with several missing feasibility elements. Improved adherence to CONSORT guidelines and enhanced transparency are needed.

**Supplementary Information:**

The online version contains supplementary material available at 10.1186/s40814-026-01859-x.

## Key messages

Issues being addressed

- Pilot and feasibility studies are crucial for advancing research, yet a historical lack of reporting guidelines has hindered standardisation.
- Consequently, inconsistencies have been noted in the quality of published literature, with reporting completeness in the areas of hip and knee arthroplasty remaining unexplored despite the field’s growth.

What we know about the topic

- Published In 2016, CONSORT extension for pilot and feasibility trials was designed to enhance reporting completeness with low adherence identified in surgery, cardiology, and pediatric nephrology.
- While factors such as industry funding, study population size, and structured abstracts have been studied for their impact on reporting completeness, findings have been inconsistent.

Key findings from this study

- Reporting completeness was low, with studies reporting an average of 55.0% of applicable CONSORT extension criteria and lacking 71.4% of key feasibility elements.
- Less complete reporting was associated with a lack of disclosed funding and more missing feasibility elements. Studies with structured abstracts, pharmacologic interventions, industry funding, and mentions of reporting guidelines showed slightly higher completeness.

Implications

- The gaps in reporting identified through this study emphasise key areas for researchers to consider when developing pilot or feasibility projects.
- This study highlights the importance of guideline awareness in enhancing research quality, supporting the need to promote and further investigate CONSORT extension adoption across fields.

## Background

When determining the viability of a clinical trial, one method many researchers consider is directing a preliminary study, termed a “pilot” or “feasibility” study. Pilot studies are conducted on smaller scales relative to their respective larger studies; the intended purpose is to appraise the methodology of the full-scale study, particularly by assessing the quality and efficiency of said methodologies and their implementations [1]. Similarly, feasibility studies are designed with the purpose of examining feasibility/viability of a main trial. Feasibility and pilot studies often act as a considerable contributing factor for successful main studies (producing studies of high-quality and validity) [1, 2]. Other reasons for conducting pilot/feasibility studies include determining an appropriate sample size, selecting necessary resources, and providing initial evidence to funding bodies in order to obtain support. Researchers also conduct pilot and feasibility studies with the goal of revealing any protocol shortcomings, thus aiding in the development of effective research protocols [2]. When creating and analysing the efficacy of research protocols, various areas of interest include measurement tools, research parameters, and sample sizes [3].

A significant challenge stems from the inconsistent reporting observed in studies labeled as “pilot” or “feasibility” [3]. There is an association between insufficient reporting and the historical lack of pilot and feasibility studies reporting guidelines [4]. In a review of pilot studies done by Kaur et al., only 58% of studies reviewed clearly stated their specific purposes of piloting; additionally, only 12% of “pilot” and “feasibility” articles progressed to a definitive clinical trial [5]. As a result of pilot/feasibility studies not following appropriate reporting guidelines (i.e. CONSORT), studies are often labeled incorrectly, not distinguished from small clinical studies, lack rationale for chosen sample sizes, and also lack appropriate objectives (i.e. feasibility indicators, efficacy indicators) [5].

A 2010 review indicated that 81% of intervention-based pilot studies were conducted with insufficient sample sizes [6]. Several systematic reviews have highlighted the lack of reporting standardisation; reviews show that more standardised reporting is necessary in order to accurately evaluate study outcomes and implications essential to fostering field progress [7]. The Consolidated Standards of Reporting Trials (CONSORT) statement, is a guideline published In 1996 for the reporting of pilot and feasibility studies; the extension was later created to improve pilot trial reporting quality [4, 8]. The CONSORT extension to pilot and feasibility trials, proposed by Eldridge et al., extends the original statement with a 26-item checklist containing items to include for effective high quality reporting [9]. Item descriptions include explanations for the inclusion of each item, each with its own description/definition. This extension, published In 2016, applies to any randomised study conducted prior to a definitive RCT (e.g. pilot and feasibility trials and studies) [9]. Several researchers have widely adopted the CONSORT extension and consequently work to improve the robustness of pilot studies in a setting of otherwise suboptimal reporting practices [8, 9].

This study focuses on pilot trials in total hip and knee arthroplasty, one of the most common inpatient surgeries and regularly performed joint replacement procedures [10]. According to the Canadian Institute for Health Information, indications included osteoarthritis, inflammatory arthritis, acute hip fracture, and infectious arthritis [11]. Similarly, Otten et al. project that the number of arthroplasties may increase by 149% and 297% for hip and knee procedures by 2030, respectively [12]. With this context in mind, relevant field research is essential to maintain quality and improve patient outcomes. As of October 2024, clinicaltrials.gov reports 204 and 432 actively recruiting clinical trials in hip and knee arthroplasties, respectively. Proper reporting and pilot study progression is necessary for timely field development. With this as the case, the primary objective of this study is to evaluate reporting completeness in hip and knee arthroplasty pilot and feasibility trials, as qualified using the CONSORT extension checklist for pilot trials [see Appendix 1 in Additional file 2]. Secondary objectives are to (1) evaluate the completeness of reporting in relation to *key feasibility items* [derived from the complete extension checklist; see Appendix 2 in Additional file 2] and (2) identify factors associated with the completeness of reporting based on the CONSORT extension using regression analysis.

## Methods

### Study design

This study involves a methodological survey of pilot and feasibility literature in hip and knee arthroplasty for reporting completeness based on the CONSORT extension [9]. The manuscript has been presented in accordance with the Preferred Reporting Items for Systematic Reviews and Meta-Analyses (PRISMA) guidelines for reporting methodology research. The protocol for this study has been published in the British Medical Journal Open, accessible at 10.1136/bmjopen-2024-085441 [13].

### Eligibility criteria

Articles were eligible for inclusion if they were described as a pilot or feasibility study in any section of the title, abstract, or main text, with the terms “study” and “trial” used interchangeably. Eligible studies must have been published in English between January 01, 2017, and December 31, 2023, in the PubMed database (via OVID). Additionally, the focus of the studies needed to be on hip and knee arthroplasty procedures with human participants, specifically examining interventions aimed at improving preoperative, procedural, or postoperative clinical outcomes. Articles performed on or involving an animal population, pending publication, or involving case reports or secondary research (review articles, meta-analyses) were excluded.

### Search strategy

We conducted a search of the PubMed database from January 01, 2017, to December 31, 2023. This date range was selected to allow for a long-term overview of CONSORT extension use from its publication In 2016, up to 2023, the year prior to study conception. Medical subject headings and keywords including “feasibility studies,” “pilot projects,” "arthroplasty, replacement knee” and “arthroplasty, replacement, hip” were used to identify relevant texts. As the CONSORT 2010 extension was created to include both pilot and feasibility RCTs, this study will also include pilot and feasibility studies. The complete PubMed search was conducted using the search strategy documented in Appendix 3 [see Additional file 2] to identify publications for review.

### Study selection and assessment

Search results were exported onto Microsoft Excel, after which duplicate entries were removed. Subsequently, a primary reviewer (Z.C.) screened all texts by title based on the inclusion and exclusion criteria to identify entries for full-text screening. During this process, a second reviewer (L.G.) screened a random sample of 50 entries to ensure consistent concept interpretation and accuracy of qualification based on eligibility criteria.

Following the screening phase, data extraction was completed by the primary reviewer, after which a sample of 50 entries was extracted by a second reviewer to further examine accuracy and quality. If the level of agreement did not meet the minimum acceptable standard of 80%, the sample would be expanded to encompass the entire screening population, which was not the case for this study. Discrepancies between the two reviewers (Z.C. and L.G.) were resolved by reaching a consensus. If deemed necessary, a third reviewer (L.T.) was available to address any persistent disagreements (however, this was not applicable at this stage and a third reviewer was not involved).

### Sample size and sampling strategy

The minimum acceptable sample size for this study was determined using the method by Isiguzo et al. [14]. This method is based on the calculation, *n* = *1.962 (P*_*0*_* (1-P*_*0*_*)/E*^*2*^*),* where *P*_*0*_ represented the prior estimate of the proportion of studies with adequate reporting and *E* was the target margin of error [see Supplementary Table S1 in Additional file 3 for further details]. Previous literature has identified a wide variation in the level of CONSORT checklist reporting observed, ranging from a minimum around 24–26% adherence up to 67–68% [15, 16]. In this case, the minimum level of observed reporting completeness was used for this study to ensure an appropriate sample size could be reasonably met with the available literature. Therefore, assuming a level of reporting completeness at 0.25 (equivalent to 10 out of 40 CONSORT items reported) based on previous literature, a 95% confidence interval, and a 5% margin of error, 147 articles are required [15, 16]. After identifying eligible studies, articles were arranged by year, and a minimum random sample of 21 studies was selected from each of the following years (2017–2023) to make up the total minimum population of 147. This aspect of the methodology was designed to ensure a holistic assessment of CONSORT 2010 extension implementation in articles since its publication In 2016.

### Data extraction and synthesis

Data extraction was completed on Microsoft Excel and retrieved study characteristics including the date of publication, the location of the pilot or feasibility self-identifiers (title, abstract, or methods), the continent of the first author, sample size used, study design, intervention category (preoperative, intraoperative, postoperative, or combined), intervention type, and source of funding. More specific elements, such as the presence of a structured abstract or any mention of a reporting guideline (e.g. CONSORT) were also noted. Journal-specific elements, such as CONSORT endorsement or the requirement of CONSORT for publication were identified on their respective websites under the author guidelines.

The CONSORT 2010 extension was used as a measure of the completeness of pilot trial reporting. Items classified as present were indicated as such by classifying them as “1.” Unreported items were assigned “0.” Elements that were not present in the publications sample were labeled as N/A. Completeness of reporting was presented as a total sum and % of applicable reported checklist items. The percentage of complete reporting was also reported (defined as reporting ≥ 75% of the applicable checklist items).

Reporting was also evaluated using a shortened triage checklist of key feasibility items derived from the CONSORT 2010 extension. Unlike the CONSORT extension, which was designed specifically for pilot and feasibility RCTs, the key feasibility items checklist covers broader categories applicable to most study designs. Among this list are elements pertaining to feasibility intent and framing, including feasibility objectives, key feasibility outcomes, and interpretation of feasibility findings. In contrast to the CONSORT extension, which focuses on the completeness of predefined reporting outcomes, this checklist assesses whether feasibility aims and considerations are explicitly explored at the study level. The list therefore provides a baseline of reporting elements that researchers can start with, and records the presence of *incomplete* reporting, or *missing* feasibility items [see Appendix 2 in Additional file 2 for more details]. Elements on the checklist were recorded in the same manner as the CONSORT 2010 extension, using “1” for criteria that were met, “0” for criteria not met, and “N/A” for items that were not applicable to the study.

### Statistical analysis and reporting

A multivariable linear regression analysis with robust (HC1) standard errors was used to evaluate factors potentially associated with the completeness of reporting, with the dependent variable defined as the percentage of applicable CONSORT extension items reported. Independent factors included: journal’s endorsement of the CONSORT statement, journal policy mandating the inclusion of CONSORT, the presence of a structured abstract, intervention type (including postoperative and pharmacologic interventions), industry funding, unreported funding sources, and poor feasibility reporting (fewer than 75% of key feasibility criteria met) [17, 18]. Additional factors such as the date of publication (> 2020), country of the first author, type of study (e.g. randomised-controlled trial) and any manuscript reference to CONSORT or a reporting guideline were also evaluated in a separate univariable analysis. All these factors were reported as descriptive statistics, and results of the regression were reported as adjusted mean differences in reporting completeness (percentage points) with a 95% confidence interval and associated *p*-values. All *p*-values were reported to 3 decimal places; *p*-values less than 0.001 were reported as *p* < 0.001. Calculations were performed using R (version 4.4.1, R Foundation for Statistical Computing, Vienna, Austria).

## Results

### Search results

**Fig. 1:**
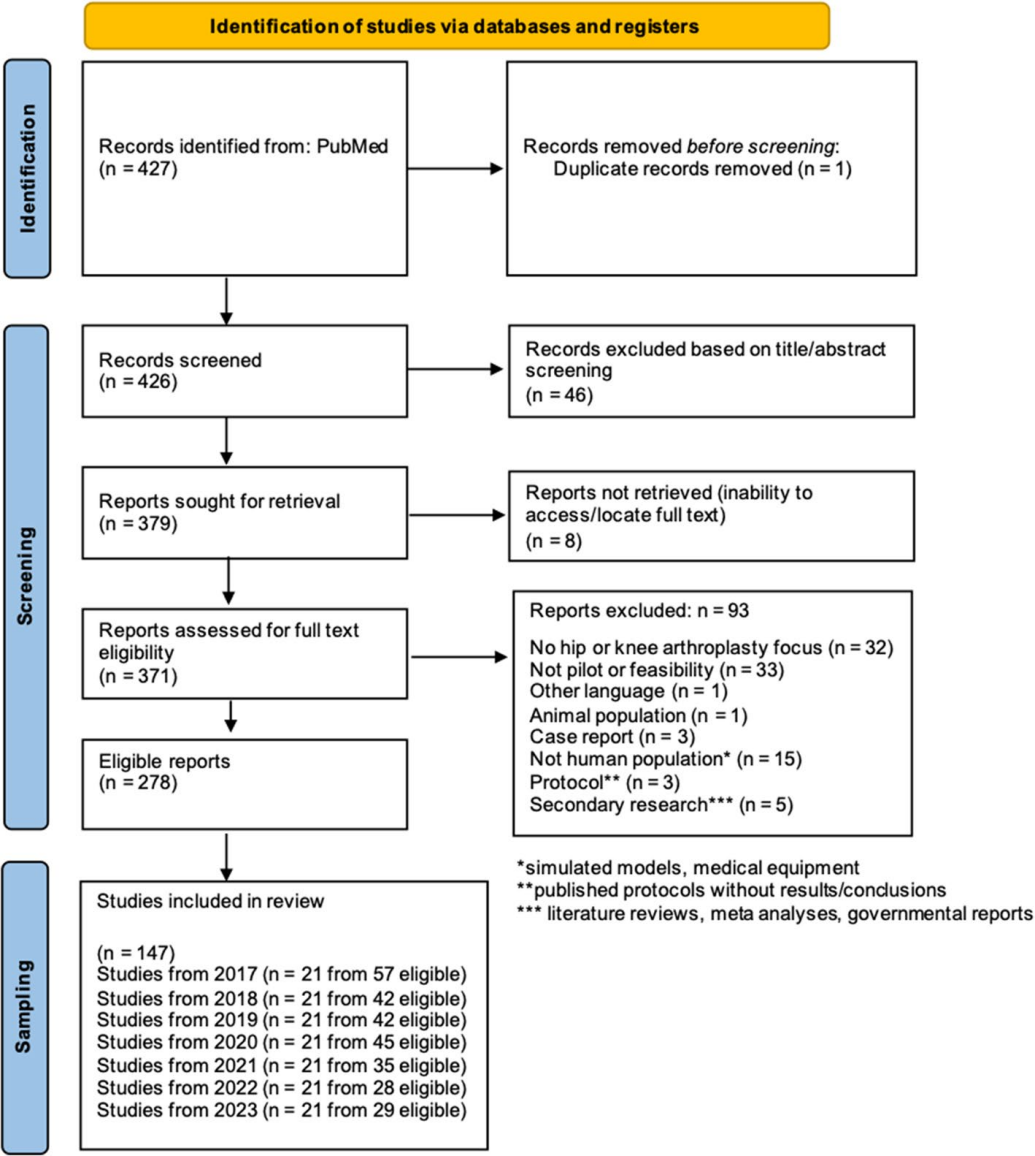
PRISMA flow chart

A total of 427 records were initially identified from the PubMed database. After screening titles, 371 records were retrieved for full-text assessment and 93 were excluded. Primary reasons for exclusion pertained to a lack of focus on hip or knee arthroplasty (*n* = 32) or a failure to identify the study as a pilot or feasibility trial (n = 31). Of the 278 eligible publications, a random sample of 21 studies was obtained from each investigated year (2017–2023) for a total of 147 included in the review. Figure 1 illustrates the Preferred Reporting Items for Systematic Reviews and Meta-Analyses (PRISMA) flow diagram with specific reasons for exclusion. The PRISMA checklist may be found under Additional file 1.

### Agreement of reviewers

Cohen’s kappa score was calculated to be 0.92 for the CONSORT extension and 0.85 for the key feasibility items checklist. These scores (> 0.8) suggest strong to almost perfect inter-rater reliability between the two reviewers for the compared entries [19].

### Study characteristics

Table 1 provides a summary of the 147 studies included in this methodological survey, evenly distributed from 2017 to 2023. However, of note, the number of eligible studies differed across years, with a larger number available from earlier dates (e.g. 57 studies from 2017) relative to later years (e.g. 29 studies from 2023). From these variable pools, 21 studies per year were randomly sampled. A majority (*n* = 111, 75.5%) were labeled as “pilot” studies, with the remainder (*n* = 35, 23.8%) identified as “feasibility” studies. This review focused on research in hip (n = 40, 27.2%) and knee arthroplasty (*n* = 78, 53.1%), with a subset of 19.7% (*n* = 29) investigating both procedures. Over half (*n* = 77, 52.4%) of the studies were led by first authors originating from Europe. Randomised controlled trials (RCTs) were the most prevalent study design (*n* = 44, 29.9%), followed by prospective cohort studies (*n* = 38, 25.9%).

**Table 1:** Characteristics of included studies (*n* = 147)

Category	Studies *n *(%)
Studies reported as pilot	111 (76)
Studies reported as feasibility	35 (24)
Identified in title	110 (75)
Identified in abstract	25 (17)
Identified elsewhere	11 (7)
Randomised studies	44 (30)
Focus of the study	
Hip arthroplasty	40 (27)
Knee arthroplasty	78 (53)
Both	29 (20)
Continent of first author	
Europe	77 (52)
America	39 (27)
Asia	25 (17)
Oceania	4 (3)
Africa	1 (1)
Study design	
RCT (randomised controlled and randomised clinical)	44 (30)
Prospective cohort	38 (26)
Non-randomised clinical trial	17 (12)
Retrospective cohort	14 (10)
Prospective cross-sectional	11 (7)
Retrospective case-control	4 (3)
Mixed methods	3 (2)
Survey	1 (1)
Other	14 (10)
Intervention type	
Preoperative	27 (18)
Intraoperative	31 (21)
Postoperative	77 (52)
Combination	10 (7)
N/A	2 (1)
Source of funding	
Governmental	40 (27)
Internal (academic)	13 (9)
Commercial	12 (8)
Other private	3 (2)
Not-for-profit	9 (6)
No funding	28 (19)
Not reported	42 (29)
Mean study population (SD) (ITT)	31 ± 20.22
Reporting guideline referenced	
CONSORT (unspecified)	15 (10)
2010 CONSORT	6 (4)
2016 CONSORT extension	3 (2)
STROBE	3 (2)
STARD	0 (0)
SRQR	0 (0)
SQUIRE	0 (0)
Other reporting guideline	1 (1)
None reported	119 (81)

Funding sources varied, with 27.2% (*n* = 40) of studies reporting funding from governmental sources, 19.0% (*n* = 28) stating no funding and 28.6% (*n* = 42) lacking funding details. Reporting guidelines were seldom referenced, with only 16.3% (*n* = 24) citing the CONSORT guidelines or its extension and 1.4% (*n *= 2) mentioning STROBE.

### Completeness of reporting – CONSORT 2010 Checklist Extension

Overall, the included studies reported an average of 55.0% (SD 17.0) of applicable CONSORT extension checklist items, with randomised controlled trials (RCTs) covering 62.5% (SD 16.5) compared to non-RCTs at 50.7% (SD 15.7). When all criteria were considered, regardless of applicability, the mean number of reported items was 14.6 (SD 6.3). Furthermore, 11.6% (*n* = 17) of all included studies reported over 75% of the applicable CONSORT extension criteria. An overview of CONSORT extension reporting is summarised in Table 2.

**Table 2:** Overview of completeness of reporting based on the CONSORT extension for pilot/feasibility trials (*n *= 147)

Overview of CONSORT extension reporting	Value	95% CI
All studies (*n* = 147)		
Mean % of applicable checklist items reported | *mean (SD)*	54.98 (16.95)	52.24, 57.72
Mean number of checklist items reported/40 | *mean (SD)*	14.56 (6.34)	13.54, 15.59
Total number of studies reporting over 75% of applicable criteria *| n (%)*	17 (11.56)	6.39, 16.73
RCTs (*n* = 53)		
Mean % of applicable checklist items reported | *mean (SD)*	62.51 (16.52)	58.06, 66.96
Mean number of checklist items reported/40 | *mean (SD)*	19.62 (6.22)	17.95, 21.30
Total number of studies reporting over 75% of applicable criteria *| n (%)*	11 (20.75)	9.84, 31.67
Non-RCTs (*n* = 94)		
Mean % of applicable checklist items reported | *mean (SD)*	50.73 (15.74)	47.55, 53.91
Mean number of checklist items reported/40 |*mean (SD)*	11.71 (4.33)	10.84, 12.59
Total number of studies reporting over 75% of applicable criteria *| n (%)*	6 (6.38)	1.44, 11.32

Among criteria applicable to over 50% of studies, the most frequently reported ones focused on pilot trial limitations (criterion 20; *n* = 131, 89.1%), details of the intervention (criterion 5; *n* = 131, 89.1%), the inclusion of a structured abstract (criterion 1b; *n* = 125, 85.0%), and the reporting of eligibility criteria (criterion 4a; *n* = 122, 83.6%). In contrast, few studies mentioned any prespecified criteria for deciding whether to proceed with a future definitive trial (criterion 6c; *n* = 8, 5.4%). Other poorly reported criteria centred on providing a rationale for conducting the pilot trial (criterion 2a; *n* = 14, 9.5%), specific pilot trial objectives (criterion 2b; *n* = 26, 17.7%), and implications for progressing the pilot to a future definitive trial (criterion 22a;* n* = 39, 26.5%). Supplementary Table S2 captures the complete summary of CONSORT extension reporting [see Additional file 3].

### Completeness of reporting – key feasibility items checklist

In contrast to the reporting of CONSORT extension items, studies lacked approximately 71.4% (SD 28.2) of applicable key feasibility elements identified on the checklist. Relative to the CONSORT extension, which was tailored for pilot and feasibility RCTs, the key feasibility items checklist encompasses broader categories relevant to various study designs. Even so, RCTs still reported more feasibility elements than non-RCTs, missing an average of 62.9% (SD 34.7) of applicable feasibility items relative to 76.1% (SD 22.6) for non-RCTs.

Criteria related to feasibility objectives and outcomes were particularly underreported. Most studies did not include sample size justifications based on feasibility objectives (main text criterion c; *n* = 133, 90.5%), criteria for determining feasibility (main text criterion b; *n* = 133, 90.5%), results of feasibility objectives in the abstract (abstract criterion a; *n* = 121, 82.3%), and conclusions relating to feasibility (abstract criterion b; *n* = 116, 78.9%). However, most manuscripts did report the results of pilot or feasibility trials (scope criterion a; *n* = 0, 0% did not report), included the terms “feasibility” or “pilot” as identifiers in the title (title criterion a; *n* = 38, 25.9% did not report) featured some primary objectives on the assessment of feasibility (main text criterion a; *n* = 102, 69.4% did not report), and addressed uncertainties regarding feasibility (key messages criteria a; *n* = 85, 57.8% did not report). Table 3 captures the full reporting overview.

**Table 3:** Completeness of reporting based on the key feasibility items checklist (*n *= 147)

Category	Feasibility criteria	Number of studies (*n*) meeting criteria	% applicable studies meeting criteria (95% CI)
All studies (*n* = 147)			
Scope	a. The manuscript appears to be out of scope: it is not reporting the results of pilot, feasibility or proof-of-concept	0	0, (0.00, 3.00)
Title	a. The title does NOT have the terms “feasibility” or “pilot” OR “proof-of-concept”	38	25.9 (18.77, 32.93)
Abstract	a. The abstract does NOT report the feasibility objectives results	121	84 (78.11, 85.95)
	b. The conclusions do NOT relate to feasibility	116	80 (73.53, 86.47)
Key messages	a. At least 1 of 3 key feasibility items are NOT met: ● What uncertainties existed regarding the feasibility? ● What are the key findings on feasibility? ● What are the implications of the findings for the design of the main study?	71	48 (40.22, 56.38)
Main text	a. Objectives: The primary objectives are NOT on feasibility assessment	102	69 (61.94, 76.84)
	b. Feasibility outcome and Progression criteria: The feasibility outcomes are NOT specified along with corresponding criteria for determining success of feasibility	133	90 (85.73, 95.22)
	c. Sample size: The sample size justification is NOT based on feasibility objectives	133	90 (85.73, 95.22)
	d. Analysis: The analysis description does NOT include analysis of feasibility outcomes	114	78 (70.81, 84.30)
	e. Results: The feasibility results are NOT reported	109	74 (67.07, 81.23)
Quality	a Overall quality of the manuscript does NOT appear to be of high quality (based on the abstract, nature of the main text)	101	69 (61.21, 76.20)
Randomised controlled trials (*n* = 53)			
Scope	a. The manuscript appears to be out of scope: it is not reporting the results of pilot, feasibility or proof-of-concept	0	0, (0.00, 3.00)
Title	a. The title does NOT have the terms “feasibility” or “pilot” OR “proof-of-concept”	10	19 (8.33, 29.40)
Abstract	a. The abstract does NOT report the feasibility objectives results	36	71 (58.32, 82.86)
	b. The conclusions do NOT relate to feasibility	35	67 (54.68, 79.94)
Key messages	a. At least 1 of 3 key feasibility items are NOT met: ● What uncertainties existed regarding the feasibility? ● What are the key findings on feasibility? What are the implications of the findings for the design of the main study?	25	47 (33.73, 60.61)
Main text	a. Objectives: The primary objectives are NOT on feasibility assessment	33	62 (49.21, 75.31)
	b. Feasibility outcome and Progression criteria: The feasibility outcomes are NOT specified along with corresponding criteria for determining success of feasibility	43	81 (70.60, 91.67)
	c. Sample size: The sample size justification is NOT based on feasibility objectives	44	83 (72.91, 93.13)
	d. Analysis: The analysis description does NOT include analysis of feasibility outcomes	36	68 (55.36, 80.49)
	e. Results: The feasibility results are NOT reported	33	62 (49.21, 75.31)
Quality	a Overall quality of the manuscript does NOT appear to be of high quality (based on the abstract, nature of the main text)	30	57 (43.26, 69.95)
Non-randomised controlled trials (*n* = 94)			
Scope	a. The manuscript appears to be out of scope: it is not reporting the results of pilot, feasibility or proof-of-concept	0	0, (0.00, 3.00)
Title	a. The title does NOT have the terms “feasibility” or “pilot” OR “proof-of-concept”	28	30 (20.54, 39.03)
Abstract	a. The abstract does NOT report the feasibility objectives results	85	91 (85.73, 97.07)
	b. The conclusions do NOT relate to feasibility	81	87 (80.32, 93.87)
Key messages	a. At least 1 of 3 key feasibility items are NOT met: ● What uncertainties existed regarding the feasibility? ● What are the key findings on feasibility? What are the implications of the findings for the design of the main study?	46	49 (38.83, 59.04)
Main text	a. Objectives: The primary objectives are NOT on feasibility assessment	69	73 (64.47, 82.34)
	b. Feasibility outcome and Progression criteria: The feasibility outcomes are NOT specified along with corresponding criteria for determining success of feasibility	90	96 (91.66, 99.83)
	c. Sample size: The sample size justification is NOT based on feasibility objectives	89	95 (90.14, 99.22)
	d. Analysis: The analysis description does NOT include analysis of feasibility outcomes	78	83 (75.38, 90.58)
	e. Results: The feasibility results are NOT reported	76	81 (72.90, 88.81)
Quality	a Overall quality of the manuscript does NOT appear to be of high quality (based on the abstract, nature of the main text)	71	76 (66.84, 84.22)

### Factors associated with completeness of reporting

**Fig. 2:**
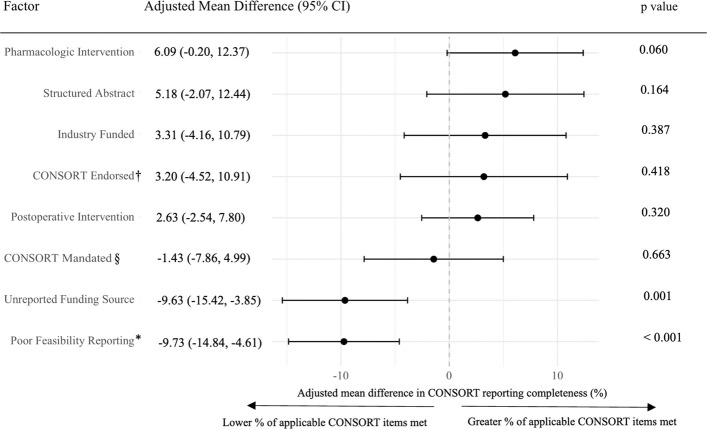
Multivariable analysis and forest plot of factors associated with completeness of reporting according to the CONSORT extension for pilot/feasibility trials. †CONSORT was mentioned or recommended in the publishing journal’s author guidelines. §The CONSORT full checklist or extension was required for manuscript submission. *Defined as meeting ≥ 75% of the items on the key feasibility checklist. Coefficient estimates represent adjusted mean differences in reporting completeness derived from a multivariable linear regression model, including the following prespecified predictors: structured abstract, pharmacologic intervention, industry funding, CONSORT endorsement (from the journal of study publication), mandated CONSORT use (required for publication), postoperative intervention, unreported funding source, and poor feasibility reporting (fewer than 75% of key feasibility items reported). Robust (HC1) standard errors were used. Multivariable analysis was performed to evaluate independent associations while accounting for potential confounding between study characteristics. Variance inflation factors (VIF) were < 2 for all predictors, indicating no evidence of problematic multicollinearity

A multivariable, linear regression analysis with robust standard errors revealed that most factors evaluated in this study had only weak associations with reporting quality. Among them, studies focusing on pharmacologic interventions were associated with an average of 6.09 percentage points higher CONSORT adherence compared with other studies, holding all other predictors constant (95%CI − 0.20, 12.37). Studies including structured abstracts were associated with a 5.2 percentage point greater reporting completeness (95%CI − 2.07, 12.44) while those with industry funding also exhibited more complete reporting practices (3.31 percentage points, 95%CI − 4.16, 10.79). Comparatively, policies mandating the use of CONSORT (− 1.43 percentage points, 95%CI − 7.86, 4.99) did not significantly impact reporting quality. These findings are showcased through a forest plot in Fig. 2, all of which did not reach statistical significance*.*

Conversely, studies with poor feasibility reporting (failing to meet ≥ 75% of key feasibility items) disclosed significantly fewer CONSORT extension criteria (− 9.73 percentage points, 95%CI − 14.84, − 4.61; *p* < 0.001). Similarly, those that did not disclose their funding source had 9.63 percentage points lower reporting completeness (95%CI − 15.42, − 3.85; *p* = 0.001). No other significant associations were identified (see Fig. 2 for reference). A further, exploratory univariable analysis of additional study characteristics (publication after 2020, originating from Europe, RCT design, mention of CONSORT, mention of any reporting guideline) revealed that conducting RCTs was associated with a 10.42 percentage points higher reporting completeness (95%CI 4.57, 16.27) and mentioning CONSORT or any reporting guideline within the manuscript was associated with 22.18 (95%CI 17.62, 26.75) and 23.4 percentage point increases (95%CI 18.67, 28.17), respectively. However, factors such as the year of publication and the country of the first author were not significantly associated with reporting quality. Completeness fluctuated across publication years without a clear trend of improvement or decline relative to 2017 [see Supplementary Table S3 in Additional file 3 for details].

## Discussion

### CONSORT extension reporting

**Fig. 3:**
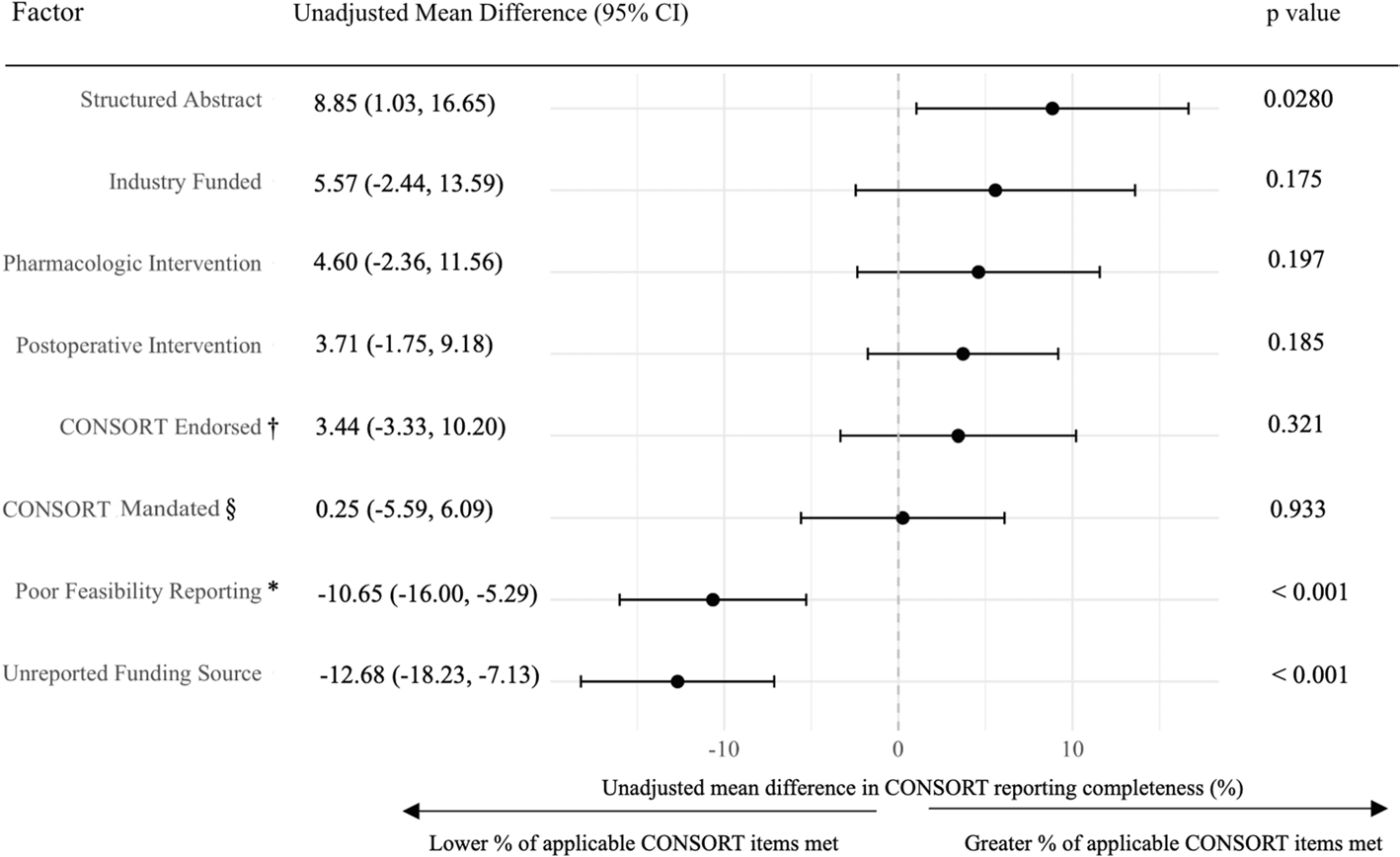
Univariable analysis and forest plot of factors associated with completeness of reporting according to the CONSORT extension for pilot/feasibility trials. †CONSORT was mentioned or recommended in the publishing journal’s author guidelines. §The CONSORT full checklist or extension was required for manuscript submission. *Defined as meeting ≥ 75% of the items on the key feasibility checklist. Coefficient estimates represent unadjusted mean differences derived from separate univariable linear regression models evaluating each predictor individually. Robust (HC1) standard errors were used

This methodological survey found that, on average, 55.0% of applicable CONSORT extension items were addressed per study, highlighting suboptimal reporting practices among pilot and feasibility trials in hip and knee arthroplasty. Despite including both RCTs and non-RCTs in this study, the level of reporting completeness here matched findings across various disciplines [20, 21]. These values ranged from 56% in surgical pilot RCTs to 68% for pilots in diabetic retinopathy [16, 20]. Nonetheless, RCTs demonstrated a greater degree of completeness relative to non-RCTs, even after adjusting for applicable items. Studies that included a pharmacologic intervention, a structured abstract, or industry funding were associated with estimated increases in 6.1, 5.2, and 3.3 percentage points, respectively, in the proportion of applicable CONSORT checklist items reported. Poor reporting was made further evident by the key feasibility items checklist, where studies failed to report 71.4% of recommended feasibility elements. Criteria pertaining specifically to determining and evaluating feasibility remained underreported, despite most manuscripts using the “pilot” or “feasibility” identifiers in their titles Fig. 3.

The reporting of basic study characteristics, such as eligibility criteria, intervention details, and research limitations, was generally adequate. These aspects are recognised across scientific literature as core manuscript elements and are consistently included in established checklists like the CONSORT 2010 checklist for RCTs or the STARD 2015 checklist for diagnostic accuracy studies [22–24]. Conversely, items specific to pilot and feasibility trials—such as criteria for progression to a full-scale trial, rationale for piloting, specific pilot objectives, and implications for future definitive trials—were underreported. This trend mirrors the findings of Bhatt et al. In 2017, highlighting minimal improvements in the quality of pilot trial reporting over the years [25]. As outlined in previous reviews, the incorrect designation of “pilot” for smaller or underpowered trials likely contributes to this discrepancy [26]. Other works were found to use “pilot” to emphasise the preliminary nature of their findings, rather than reserving it for projects designed for planning larger, main trials [3, 27]. These observations underscore the ongoing need to clarify the distinctive role of pilot/feasibility trials compared to their full-scale counterparts. Importantly, in a separate, exploratory univariable analysis, the mention of CONSORT (22.18 percentage points, 95%CI 17.62, 26.75) or another reporting guideline (23.42 percentage points, 95%CI 18.67, 28.17) within a manuscript was associated with the greatest increase in reporting completeness. With this as the case, raising awareness of the CONSORT extension among authors could streamline arthroplasty pilot literature in the same way the CONSORT checklist has improved RCT reporting [28].

As noted in previous literature reviews, a journal’s endorsement of CONSORT and the year of publication had a limited influence on reporting quality [20, 25]. These findings emphasise the importance of an author’s active engagement with the CONSORT extension, as many journal guidelines do not clearly differentiate it from the original CONSORT list for full-scale studies. Furthermore, the inclusion of a structured abstract, the use of pharmacologic interventions and the presence of an industry funding source were linked to slight increases in reporting completeness. The literature around structured abstracts has varied, with Helbach et al. suggesting that manuscripts with structured abstracts adhered more often to the PRISMA-A guidelines, while Scherer et al. found this made no difference in CONSORT reporting [29, 30]. Meanwhile, acknowledging the potential for sponsorship bias, industry-funded researchers have made efforts to actively register their trials and publish results in registries [31, 32]. Such actions may support improved reporting completeness as authors strive to demonstrate credibility and combat public perceptions of bias being reflected in their work [32]. At the same time, the industry-based environment itself can differ greatly from grant/university or self-funded settings. Here, the presence of additional reviewers, resources, or organisation protocols can not only promote study completion, but also provide researchers with additional insight into available tools such as the CONSORT extension. Finally, RCTs likely comprised a greater proportion of pharmacologic studies in this survey compared to other interventions, having been established as the gold standard of evidence in the field [33]. With CONSORT 2010 (for RCTs) being well known among researchers, even authors who are unaware of the extension may be preparing their pharmacologic studies in accordance with the precedent set by full-scale checklist. Again, the requirement of publication to a registry, such as clinicaltrials.gov, may also be more prevalent among pharmacologic studies involving increased risk and long-term monitoring relative to other intervention types.

### Key feasibility items

The key feasibility items checklist reflected a similar pattern of incomplete reporting. Although designed for all study types (unlike the CONSORT extension, which targets pilot and feasibility RCTs), RCTs demonstrated greater reporting completeness compared to non-RCTs. This difference likely stems from the RCTs’ status as the highest level of evidence, where they have become the topic of widespread standardisation across disciplines [34]. Pilot RCTs, informed by these standards, may benefit from more structured designs. Nonetheless, the feasibility checklist may be particularly useful for non-RCT pilots, given that a higher proportion of its criteria are applicable across different study designs (mean of 100% applicable criteria) when compared to the CONSORT extension (mean of 30% applicable criteria). Additionally, most criteria that focused specifically on defining, measuring, and reporting feasibility outcomes were poorly reported, aligning with findings from the CONSORT extension. The similar patterns in feasibility and CONSORT extension reporting likely reflect the conceptual overlap between the two frameworks. Greater engagement with feasibility-specific considerations may therefore be associated with improved CONSORT reporting quality, whereas poor feasibility reporting was associated with a 9.73 percentage point reduction in compliance (95%CI − 14.84, − 4.61; *p* < 0.001).

As suggested by similar patterns in nursing intervention research and exercise prehabilitation, the misidentification of this study design may also contribute to these observations [35, 36]. For instance, hypothesis testing was found to be the primary focus of “feasibility literature” in nursing interventions, rather than the investigation of study acceptability [35]. Consequently, incomplete reporting on core feasibility parameters, such as retention rates and clinical generalisability, limits the ability to interpret true intervention feasibility and translate findings to future studies [34].

### Strengths and limitations

This study assessed reporting completeness using both the CONSORT 2016 extension and the key feasibility items checklist, allowing for a comprehensive evaluation across different study types. The stratified random sampling approach ensured an equal distribution of studies across the years investigated, producing an objective view of reporting trends over time. This was in combination with a multivariable linear regression with robust (HC1) standard errors, which helped to identify factors influencing reporting quality. However, while temporal coverage was improved, this approach may introduce selection bias through the disproportionate sampling of years with lower publication volumes, rather than reflecting the underlying distribution of eligible studies (e.g. 28 studies In 2022 vs 57 In 2017).

Other study limitations stemmed from the sample size, potential sampling bias, and outcome categorisation. The sample size of 147 was calculated using the lowest published adherence rate to CONSORT extension (25%), rendering it potentially insufficient [16]. To achieve a 5% margin of error based on the actual mean percentage of CONSORT items reported in this study, a sample size of 194 would have been more appropriate. Furthermore, all studies were sourced from a single database, PubMed, and the majority came from European countries, introducing potential selection bias. Lastly, this study did not conduct independent subgroup analyses for hip and knee arthroplasty trials, which were assessed together.

Future methodological work may benefit from incorporating broader searches across multiple major databases, including Scopus, CINAHL and Web of Science, reducing reliance on a single database. From this population, a proportional or weighted stratified sampling approach may better reflect the true distribution of eligible studies across years. Pre-specification of clinically relevant subgroups may also enable more thorough assessments and comparisons (e.g. hip versus knee arthroplasty trials).

### Deviations from the protocol

The previously published protocol has been modified for this study to narrow the focus of the manuscript and explore reporting completeness in depth [13]. A separate review is currently planned for the exploration of additional factors, such as the prevalence of misleading reporting practices in arthroplasty literature. The screening sample specified in “study selection” section was additionally increased from the previous value of 20 to a greater subset of 50 entries to allow for improved accuracy and comparison across reviewers.

### Implications for practice

Between 2000 and 2019, the average volume of total hip (THA) and total knee arthroplasty (TKA) reportedly increased by 177% and 156%, respectively, according to US Medicare data [37]. Similar growth is projected across OECD countries over the next 35 years [38]. This rising demand stresses the importance of well-designed and efficient research, to accommodate such exponential growth. Here, projects focused on improving surgical techniques, diagnostic procedures, pharmacologic interventions, and supportive treatment are critical to meeting these needs and ensuring optimal patient outcomes. Unfortunately, the completeness of pilot trials intended to facilitate the development of such research remains limited.

## Recommendations

Given that factors such as the mention of reporting guidelines, structured abstracts, and industry funding are associated with more complete reporting, promoting awareness of reporting guidelines and encouraging trial registration could improve research quality. This is important both at a journal and author level. In an assessment of the CONSORT 2010 full checklist (for RCTs) Turner et al. note that journals continue to send unclear messages regarding endorsement, highlighting the need for further action surrounding CONSORT use [39]. Notably, although leading orthopedic journals, such as *The Bone and Joint Journal, The Journal of Bone and Joint Surgery,* and *Clinical Orthopedics and Related Research*, all clearly reference the CONSORT statement in their author guidelines, they do not distinguish pilot and feasibility trials separately or directly reference the extension.

With this as the case, the inclusion of a more distinct category for pilot and feasibility trials within their guidelines and submission process. This section may outline targeted requirements and objectives for these study types and help clarify the distinction between the full CONSORT checklist and its extension. Authors submitting such projects may be required to explicitly classify their work as either a pilot or feasibility study according to journal-defined criteria or standards. In this context, journals may play a key role in clarifying the distinct roles of these study types, with pilot trials aimed primarily at testing trial processes and methodology in preparation for a definitive project, and feasibility studies evaluating the practical and operational viability of implementing an intervention or design. This is in line with the work led by Pearson et al., which noted that further guidance on *when* to conduct feasibility or pilot studies can help to ensure projects are initiated with the appropriate goals in mind [40].

Additionally, stricter enforcement of CONSORT extension adherence at the journal level may be necessary, rather than simply listing the checklist as a recommendation. Beyond passive endorsement through an editorial check, having the extension explicitly listed as a mandatory requirement with pilot RCT submissions and verified during the editorial process would integrate it formally into the peer-review process. Incorporating the CONSORT extension into structured peer-review forms and concurrent training modules may further ensure both consistent implementation and understanding of the checklist and its relevance. Providing authors with tools such as the EQUATOR network’s structured training modules may further support awareness. (https://www.equator-network.org/toolkits/using-guidelines-in-journals/reporting-guidelines-and-journals-fact-fiction/).

## Conclusion

Overall, the completeness of reporting in pilot and feasibility trials for hip and knee arthroplasty is suboptimal, as indicated by the CONSORT extension and key feasibility checklists. Increasing awareness of reporting guidelines, using structured abstracts and registering study data may help to enhance reporting practices. Moving forward, future research could explore the field’s awareness of the CONSORT extension and develop strategies to improve adherence. To inform relevant planning, surveys aimed at investigating perspectives on pilot and feasibility trials should also be conducted, helping to identify and address current gaps in understanding. Additionally, further investigation into the prevalence of biased reporting and its influencing factors in hip and knee arthroplasty trials would be valuable.

## Supplementary Information

Additional file 1. PRISMA checklist 

Additional file 2. Appendix 1: CONSORT 2010 statement: extension to randomised pilot and feasibility trials. Appendix 2: Key feasibility items checklist. Appendix 3: PubMed search strategy, including terms and keywords used 

Additional file 3. Supplementary Table S1: Sample size calculation table based on a 95% confidence interval. Supplementary Table S2: Complete overview of reporting based on the CONSORT extension for pilot and feasibility trials. Supplementary Table S3: Univariable analysis of additional factors associated with completeness of reporting according to the CONSORT extension for pilot and feasibility trials 

## Data Availability

The datasets used and/or analysed during the current study are available from the corresponding author on reasonable request.
