# Supplementary material for: The reporting of CONSORT items and key feasibility elements in pilot and feasibility studies in hip and knee arthroplasty is suboptimal: a methodological survey
Source: Pilot Feasibility Stud. 2026 Jun 4;12:114. doi: 10.1186/s40814-026-01859-x (PMC13466081; doi:10.1186/s40814-026-01859-x)
Supplement: Supplementary file 1 [file 40814_2026_1859_MOESM2_ESM.pdf]

Appendix

Appendix A. CONSORT 2010 statement: extension to randomised pilot and feasibility trials[9]

Section	Item	Description	Present (Y/N)	Score (1 or 0)
**Title and abstract**	1a	Identification of study as randomized pilot or feasibility trial		
	1b	Structured summary of pilot trial design, methods, results, and conclusions (for specific guidance see CONSORT abstract extension for pilot trials)		
**Background and objectives**	2a	Scientific background and explanation of rationale for future definitive trial, and reasons for randomized pilot trial		
	2b	Specific objectives or research questions for pilot trial		
**Trial design**	3a	Description of pilot trial design (such as parallel, factorial) including allocation ratio		
	3b	Important changes to methods after pilot trial commencement (such as eligibility criteria), with reasons		
**Participants**	4a	Eligibility criteria for participants		
	4b	Settings and locations where the data were collected		

	4c	How participants were identified and consented
**Interventions**	5	The interventions for each group with sufficient details to allow replication, including how and when they were actually administered
**Outcomes**	6a	Completely defined prespecified assessments or measurements to address each pilot trial objective specified in 2b, including how and when they were assessed
	6b	Any changes to pilot trial assessments or measurements after the pilot trial commenced, with reasons
	6c	If applicable, prespecified criteria used to judge whether, or how, to proceed with future definitive trial
**Sample size**	7a	Rationale for numbers in the pilot trial
	7b	When applicable, explanation of any interim analyses and stopping guidelines
**Sequence generation**	8a	Method used to generate the random allocation sequence
	8b	Type of randomization(s); details of any restriction (such as blocking and block size)

**Allocation concealment mechanism**	9	Mechanism used to implement the random allocation sequence (such as sequentially numbered containers), describing any steps taken to conceal the sequence until interventions were assigned
**Implementation**	10	Who generated the random allocation sequence, who enrolled participants, and who assigned participants to interventions
**Blinding**	11a	If done, who was blinded after assignment to interventions (for example, participants, care providers, those assessing outcomes) and how
	11b	If relevant, description of the similarity of interventions
**Statistical methods**	12	Methods used to address each pilot trial objective whether qualitative or quantitative
**Participant flow (diagram strongly recommended)**	13a	For each group, the numbers of participants who were approached and/or assessed for eligibility, randomly assigned, received intended treatment, and were assessed for each objective
	13b	For each group, losses and exclusions after randomization, together with reasons
**Recruitment**	14a	Dates defining the periods of recruitment and follow-up

	14b	Why the pilot trial ended or was stopped
**Baseline data**	15	A table showing baseline demographic and clinical characteristics for each group
**Numbers analyzed**	16	For each objective, number of participants (denominator) included in each analysis. If relevant, these numbers should be by randomized group
**Outcomes and estimation**	17	For each objective, results including expressions of uncertainty (such as 95% confidence interval) for any estimates. If relevant, these results should be by randomized group
**Ancillary analyses**	18	Results of any other analyses performed that could be used to inform the future definitive trial
**Harms**	19	All important harms or unintended effects in each group (for specific guidance see CONSORT for harms)
	19a	If relevant, other important unintended consequences
**Limitations**	20	Pilot trial limitations, addressing sources of potential bias and remaining uncertainty about feasibility

**Generalizability**	21	Generalisability (applicability) of pilot trial methods and findings to future definitive trial and other studies
**Interpretation**	22	Interpretation consistent with pilot trial objectives and findings, balancing potential benefits and harms, and considering other relevant evidence
	22a	Implications for progression from pilot to future definitive trial, including any proposed amendments
**Registration**	23	Registration number for pilot trial and name of trial registry
**Protocol**	24	Where the pilot trial protocol can be accessed, if available
**Funding**	25	Sources of funding and other support (such as supply of drugs), role of funders
**Ethics**	26	Ethical approval or approval by research review committee, confirmed with reference number

***We strongly recommend reading this statement in conjunction with the CONSORT 2010, extension to randomised pilot and feasibility trials, Explanation and Elaboration for important clarifications on all the items. If relevant, we also recommend reading CONSORT extensions for cluster randomised trials, non-inferiority and equivalence trials, non-pharmacological treatments, herbal interventions, and*

pragmatic trials. Additional extensions are forthcoming: for those and for up-to-date references relevant to this checklist, see www.consort-statement.org.

Appendix B. Key Feasibility Items Checklist (Triage Checklist)

Item	Description	Check all that apply
**Scope**	The manuscript appears to be out of scope: it is not reporting the results of pilot, feasibility or proof-of-concept	
**Title**	The title does NOT have the terms "feasibility" or "pilot" OR "proof-of-concept"	
**Abstract**	1.The abstract does NOT report the feasibility objectives results.<br>2. The conclusions do NOT relate to feasibility	
**Key messages on feasibility**	The three key messages on feasibility are missing:<br>a. What uncertainties existed regarding the feasibility?<br>b. What are the key findings on feasibility?<br>c. What are the implications of the findings for the design of the main study?	
**Main text**	The main text does not have the key elements on feasibility<br>a. Objectives: The primary objectives are NOT on feasibility assessment<br>b. Feasibility outcome and Progression criteria: The feasibility outcomes are NOT specified along with corresponding criteria for determining success of	

feasibility

c. Sample size: The sample size justification is NOT based on feasibility objectives

d. Analysis: The analysis description does NOT include analysis of feasibility outcomes

e. Results: The feasibility results are NOT reported

**Overall quality**	Overall quality of the manuscript does NOT appear to be of high quality (based on the abstract, nature of the main text)

Appendix C. PubMed Search Strategy

	Search terms	Results
**#1**	("Feasibility studies"[MeSH Terms] OR "feasibility project*"[Text Word] OR "feasibility trial*"[Text Word] OR "feasibility trials"[Text Word] OR "feasibility stud*"[Text Word]) AND (2017:2023[pdat])	36,414
**#2**	("Pilot projects"[MeSH Terms] OR "pilot project*"[Text Word] OR "Pilot projects"[Text Word] OR "pilot trial*"[Text Word] OR "pilot trials"[Text Word] OR "pilot stud*"[Text Word]) AND (2017:2023[pdat])	70,673
**#3**	("arthroplasty, replacement, knee"[MeSH Terms] OR "knee replacement*"[Text Word] OR "knee replacements"[Text Word] OR "knee arthroplast*"[Text Word]) AND (2017:2023[pdat])	22,216

**#4**	("arthroplasty, replacement, hip"[MeSH Terms] OR "hip replacement*"[Text Word] OR "hip replacements"[Text Word] OR "hip arthroplast*"[Text Word]) AND (2017:2023[pdat])	20,347
**#5**	#1 OR #2	102,447
**#6**	#3 OR #4	37,735
**#7**	#5 AND #6	426
