# Supplementary material for: The reporting of CONSORT items and key feasibility elements in pilot and feasibility studies in hip and knee arthroplasty is suboptimal: a methodological survey
Source: Pilot Feasibility Stud. 2026 Jun 4;12:114. doi: 10.1186/s40814-026-01859-x (PMC13466081; doi:10.1186/s40814-026-01859-x)
Supplement: Supplementary file 3 [file 40814_2026_1859_MOESM3_ESM.docx]

**Supplemental Table 1:** Sample size calculation based on a 95% confidence interval

| **P0** | | | | | | | | | | | |  |
| --- | --- | --- | --- | --- | --- | --- | --- | --- | --- | --- | --- | --- |
|  |  | **0.25** | **0.3** | **0.35** | **0.4** | **0.45** | **0.5** | **0.55** | **0.6** | **0.65** | **0.7** | |
| **E** | 0.01 | 3679 | 4120 | 4464 | 4709 | 4856 | 4905 | 4856 | 4709 | 4464 | 4120 | |
|  | 0.05 | 147 | 165 | 179 | 188 | 194 | 196 | 194 | 188 | 179 | 165 | |
|  | 0.1 | 37 | 41 | 45 | 47 | 49 | 49 | 49 | 47 | 45 | 41 | |

**Supplemental Table 2**: Reporting Based on the CONSORT Extension for Pilot/Feasibility Trials (n = 147)

| **CONSORT extension criteria** | **n studies**  **reporting**  **criteria** | **%**  **applicable**  **reporting**  **criteria** | **95% CI** |
| --- | --- | --- | --- |
| **All studies (n = 147)** | | | |
| **1a.** Identification of study as randomized pilot or feasibility trial | 103 | 70.07 | 62.66, 77.47 |
| **1b.** Structured summary of pilot trial design, methods, results, and conclusions | 125 | 85.03 | 79.27, 90.80 |
| **2a.** Scientific background and explanation of rationale for future definitive trial, and reasons for randomized pilot trial | 14 | 9.52 | 4.78, 14.27 |
| **2b.** Specific objectives or research questions for pilot trial | 26 | 17.69 | 11.52, 23.86 |
| **3a.** Description of pilot trial design (such as parallel, factorial) including allocation ratio | 19 | 36.54 | 28.75, 44.32 |
| **3b.** Important changes to methods after pilot trial commencement (such as eligibility criteria), with reasons | 10 | 100.00 | 100.00, 100.00 |
| **4a.** Eligibility criteria for participants | 122 | 83.56 | 77.57, 89.55 |
| **4b.** Settings and locations where the data were collected | 99 | 67.35 | 59.77, 74.93 |
| **4c.** How participants were identified and consented | 92 | 63.45 | 55.66, 71.23 |
| **5.** The interventions for each group with sufficient details to allow replication, including how and when they were actually administered | 131 | 89.12 | 84.08, 94.15 |
| **6a.** Completely defined prespecified assessments or measurements to address each pilot trial objective specified in 2b, including how and when they were assessed | 21 | 77.78 | 71.06, 84.50 |
| **6b.** Any changes to pilot trial assessments or measurements after the pilot trial commenced, with reasons | 8 | 100.00 | 100.00, 100.00 |
| **6c**. If applicable, prespecified criteria used to judge whether, or how, to proceed with future definitive trial | 8 | 5.44 | 1.78, 9.11 |
| **7a.** Rationale for numbers in the pilot trial | 49 | 33.33 | 25.71, 40.95 |
| **7b.** When applicable, explanation of any interim analyses and stopping guidelines | 1 | 20.00 | 13.53, 26.47 |
| **8a.** Method used to generate the random allocation sequence | 38 | 71.70 | 64.42, 78.98 |
| **8b.** Type of randomization(s); details of any restriction (such as blocking and block size) | 21 | 39.62 | 31.72, 47.53 |
| **9.** Mechanism used to implement the random allocation sequence (such as sequentially numbered containers), describing any steps taken to conceal the sequence until interventions were assigned | 18 | 33.96 | 26.31, 41.62 |
| **10.** Who generated the random allocation sequence, who enrolled participants, and who assigned participants to interventions | 20 | 37.74 | 29.90, 45.57 |
| **11a.** If done, who was blinded after assignment to interventions (for example, participants, care providers, those assessing outcomes) and how | 28 | 70.00 | 62.59, 77.41 |
| **11b.** If relevant, description of the similarity of interventions | 27 | 65.85 | 58.19, 73.52 |
| **12.** Methods used to address each pilot trial objective whether qualitative or quantitative | 18 | 66.67 | 59.05, 74.29 |
| **13a.** For each group, the numbers of participants who were approached and/or assessed for eligibility, randomly assigned, received intended treatment, and were assessed for each objective | 100 | 68.03 | 60.49, 75.57 |
| **13b.** For each group, losses and exclusions after randomization, together with reasons | 72 | 82.76 | 76.65, 88.86 |
| **14a.** Dates defining the periods of recruitment and follow-up | 107 | 73.29 | 66.14, 80.44 |
| **14b.** Why the pilot trial ended or was stopped | 5 | 71.43 | 64.13, 78.73 |
| **15.** A table showing baseline demographic and clinical characteristics for each group | 112 | 76.19 | 69.31, 83.08 |
| **16.** For each objective, number of participants (denominator) included in each analysis. If relevant, these numbers should be by randomized group | 93 | 64.14 | 56.39, 71.89 |
| **17.** For each objective, results including expressions of uncertainty (such as 95 confidence interval) for any estimates. If relevant, these results should be by randomized group | 76 | 51.70 | 43.62, 59.78 |
| **18.** Results of any other analyses performed that could be used to inform the future definitive trial | 32 | 94.12 | 90.31, 97.92 |
| **19.** All-important harms or unintended effects in each group (for specific guidance see CONSORT for harms) | 61 | 85.92 | 80.29, 91.54 |
| **19a.** If relevant, other important unintended consequences | 13 | 100.00 | 100.00, 100.00 |
| **20.** Pilot trial limitations, addressing sources of potential bias and remaining uncertainty about feasibility | 131 | 89.12 | 84.08, 94.15 |
| **21.** Generalisability (applicability) of pilot trial methods and findings to future definitive trial and other studies | 54 | 36.73 | 28.94, 44.53 |
| **22.** Interpretation consistent with pilot trial objectives and findings, balancing potential benefits and harms, and considering other relevant evidence | 26 | 96.30 | 93.24, 99.35 |
| **22a.** Implications for progression from pilot to future definitive trial, including any proposed amendments | 39 | 26.53 | 19.39, 33.67 |
| **23.** Registration number for pilot trial and name of trial registry | 64 | 43.54 | 35.52, 51.55 |
| **24.** Where the pilot trial protocol can be accessed, if available | 22 | 75.86 | 68.94, 82.78 |
| **25.** Sources of funding and other support (such as supply of drugs), role of funders | 114 | 77.55 | 70.81, 84.30 |
| **26.** Ethical approval or approval by research review committee, confirmed with reference number | 59 | 40.97 | 33.02, 48.92 |
| **Randomized controlled trials (n = 53)** | | | |
| **1a.** Identification of study as randomized pilot or feasibility trial | 40 | 75.47 | 63.89, 87.06 |
| **1b.** Structured summary of pilot trial design, methods, results, and conclusions | 47 | 88.68 | 80.15, 97.21 |
| **2a.** Scientific background and explanation of rationale for future definitive trial, and reasons for randomized pilot trial | 5 | 9.43 | 1.56, 17.30 |
| **2b.** Specific objectives or research questions for pilot trial | 14 | 26.42 | 14.55, 38.28 |
| **3a.** Description of pilot trial design (such as parallel, factorial) including allocation ratio | 19 | 36.54 | 23.57, 49.50 |
| **3b.** Important changes to methods after pilot trial commencement (such as eligibility criteria), with reasons | 4 | 100.00 | 100.00, 100.00 |
| **4a.** Eligibility criteria for participants | 52 | 98.11 | 94.45, 101.78 |
| **4b.** Settings and locations where the data were collected | 38 | 71.70 | 59.57, 83.83 |
| **4c.** How participants were identified and consented | 39 | 75.00 | 63.34, 86.66 |
| **5.** The interventions for each group with sufficient details to allow replication, including how and when they were actually administered | 51 | 96.23 | 91.10, 101.36 |
| **6a.** Completely defined prespecified assessments or measurements to address each pilot trial objective specified in 2b, including how and when they were assessed | 11 | 73.33 | 61.43, 85.24 |
| **6b.** Any changes to pilot trial assessments or measurements after the pilot trial commenced, with reasons | 7 | 100.00 | 100.00, 100.00 |
| **6c**. If applicable, prespecified criteria used to judge whether, or how, to proceed with future definitive trial | 4 | 7.55 | 0.44, 14.66 |
| **7a.** Rationale for numbers in the pilot trial | 29 | 54.72 | 41.32, 68.12 |
| **7b.** When applicable, explanation of any interim analyses and stopping guidelines | 1 | 100.00 | 100.00, 100.00 |
| **8a.** Method used to generate the random allocation sequence | 38 | 71.70 | 59.57, 83.83 |
| **8b.** Type of randomization(s); details of any restriction (such as blocking and block size) | 21 | 39.62 | 26.45, 52.79 |
| **9.** Mechanism used to implement the random allocation sequence (such as sequentially numbered containers), describing any steps taken to conceal the sequence until interventions were assigned | 18 | 33.96 | 21.21, 46.71 |
| **10.** Who generated the random allocation sequence, who enrolled participants, and who assigned participants to interventions | 20 | 37.74 | 24.69, 50.79 |
| **11a.** If done, who was blinded after assignment to interventions (for example, participants, care providers, those assessing outcomes) and how | 28 | 70.00 | 57.66, 82.34 |
| **11b.** If relevant, description of the similarity of interventions | 27 | 65.85 | 53.09, 78.62 |
| **12.** Methods used to address each pilot trial objective whether qualitative or quantitative | 12 | 80.00 | 69.23, 90.77 |
| **13a.** For each group, the numbers of participants who were approached and/or assessed for eligibility, randomly assigned, received intended treatment, and were assessed for each objective | 37 | 69.81 | 57.45, 82.17 |
| **13b.** For each group, losses and exclusions after randomization, together with reasons | 38 | 77.55 | 66.32, 88.78 |
| **14a.** Dates defining the periods of recruitment and follow-up | 39 | 73.58 | 61.72, 85.45 |
| **14b.** Why the pilot trial ended or was stopped | 5 | 71.43 | 59.27, 83.59 |
| **15.** A table showing baseline demographic and clinical characteristics for each group | 46 | 86.79 | 77.68, 95.91 |
| **16.** For each objective, number of participants (denominator) included in each analysis. If relevant, these numbers should be by randomized group | 36 | 67.92 | 55.36, 80.49 |
| **17.** For each objective, results including expressions of uncertainty (such as 95 confidence interval) for any estimates. If relevant, these results should be by randomized group | 31 | 58.49 | 45.22, 71.76 |
| **18.** Results of any other analyses performed that could be used to inform the future definitive trial | 17 | 100.00 | 100.00, 100.00 |
| **19.** All-important harms or unintended effects in each group (for specific guidance see CONSORT for harms) | 29 | 90.63 | 82.78, 98.47 |
| **19a.** If relevant, other important unintended consequences | 6 | 100.00 | 100.00, 100.00 |
| **20.** Pilot trial limitations, addressing sources of potential bias and remaining uncertainty about feasibility | 50 | 94.34 | 88.12, 100.56 |
| **21.** Generalisability (applicability) of pilot trial methods and findings to future definitive trial and other studies | 23 | 43.40 | 30.05, 56.74 |
| **22.** Interpretation consistent with pilot trial objectives and findings, balancing potential benefits and harms, and considering other relevant evidence | 15 | 100.00 | 100.00, 100.00 |
| **22a.** Implications for progression from pilot to future definitive trial, including any proposed amendments | 21 | 39.62 | 26.45, 52.79 |
| **23.** Registration number for pilot trial and name of trial registry | 40 | 75.47 | 63.89, 87.06 |
| **24.** Where the pilot trial protocol can be accessed, if available | 15 | 75.00 | 63.34, 86.66 |
| **25.** Sources of funding and other support (such as supply of drugs), role of funders | 42 | 79.25 | 68.33, 90.16 |
| **26.** Ethical approval or approval by research review committee, confirmed with reference number | 25 | 48.08 | 34.63, 61.53 |
| **Non-randomized controlled trials (n = 94)** | | | |
| **1a.** Identification of study as randomized pilot or feasibility trial | 63 | 67.02 | 57.52, 76.53 |
| **1b.** Structured summary of pilot trial design, methods, results, and conclusions | 78 | 82.98 | 75.38, 90.58 |
| **2a.** Scientific background and explanation of rationale for future definitive trial, and reasons for randomized pilot trial | 9 | 9.57 | 3.63, 15.52 |
| **2b.** Specific objectives or research questions for pilot trial | 12 | 12.77 | 6.02, 19.51 |
| **3a.** Description of pilot trial design (such as parallel, factorial) including allocation ratio | 0 | 0.00 | 0.00, 0.00 |
| **3b.** Important changes to methods after pilot trial commencement (such as eligibility criteria), with reasons | 6 | 100.00 | 100.00, 100.00 |
| **4a.** Eligibility criteria for participants | 70 | 75.27 | 66.55, 83.99 |
| **4b.** Settings and locations where the data were collected | 61 | 64.89 | 55.24, 74.54 |
| **4c.** How participants were identified and consented | 53 | 56.99 | 46.98, 67.00 |
| **5.** The interventions for each group with sufficient details to allow replication, including how and when they were actually administered | 80 | 85.11 | 77.91, 92.30 |
| **6a.** Completely defined prespecified assessments or measurements to address each pilot trial objective specified in 2b, including how and when they were assessed | 10 | 83.33 | 75.80, 90.87 |
| **6b.** Any changes to pilot trial assessments or measurements after the pilot trial commenced, with reasons | 1 | 100.00 | 100.00, 100.00 |
| **6c**. If applicable, prespecified criteria used to judge whether, or how, to proceed with future definitive trial | 4 | 4.26 | 0.17, 8.34 |
| **7a.** Rationale for numbers in the pilot trial | 20 | 21.28 | 13.00, 29.55 |
| **7b.** When applicable, explanation of any interim analyses and stopping guidelines | 0 | 0.00 | 0.00, 0.00 |
| **8a.** Method used to generate the random allocation sequence | 0 | 0.00 | 0.00, 0.00 |
| **8b.** Type of randomization(s); details of any restriction (such as blocking and block size) | 0 | 0.00 | 0.00, 0.00 |
| **9.** Mechanism used to implement the random allocation sequence (such as sequentially numbered containers), describing any steps taken to conceal the sequence until interventions were assigned | 0 | 0.00 | 0.00, 0.00 |
| **10.** Who generated the random allocation sequence, who enrolled participants, and who assigned participants to interventions | 0 | 0.00 | 0.00, 0.00 |
| **11a.** If done, who was blinded after assignment to interventions (for example, participants, care providers, those assessing outcomes) and how | 0 | 0.00 | 0.00, 0.00 |
| **11b.** If relevant, description of the similarity of interventions | 0 | 0.00 | 0.00, 0.00 |
| **12.** Methods used to address each pilot trial objective whether qualitative or quantitative | 6 | 50.00 | 39.89, 60.11 |
| **13a.** For each group, the numbers of participants who were approached and/or assessed for eligibility, randomly assigned, received intended treatment, and were assessed for each objective | 63 | 67.02 | 57.52, 76.53 |
| **13b.** For each group, losses and exclusions after randomization, together with reasons | 34 | 89.47 | 83.27, 95.68 |
| **14a.** Dates defining the periods of recruitment and follow-up | 68 | 73.12 | 64.16, 92.08 |
| **14b.** Why the pilot trial ended or was stopped | 0 | 0.00 | 0.00, 0.00 |
| **15.** A table showing baseline demographic and clinical characteristics for each group | 66 | 70.21 | 60.97, 79.46 |
| **16.** For each objective, number of participants (denominator) included in each analysis. If relevant, these numbers should be by randomized group | 57 | 61.96 | 52.14, 71.77 |
| **17.** For each objective, results including expressions of uncertainty (such as 95 confidence interval) for any estimates. If relevant, these results should be by randomized group | 45 | 47.87 | 37.77, 57.97 |
| **18.** Results of any other analyses performed that could be used to inform the future definitive trial | 15 | 88.24 | 81.72, 94.75 |
| **19.** All-important harms or unintended effects in each group (for specific guidance see CONSORT for harms) | 32 | 82.05 | 74.29, 89.81 |
| **19a.** If relevant, other important unintended consequences | 7 | 100.00 | 100.00, 100.00 |
| **20.** Pilot trial limitations, addressing sources of potential bias and remaining uncertainty about feasibility | 81 | 86.17 | 79.19, 93.15 |
| **21.** Generalisability (applicability) of pilot trial methods and findings to future definitive trial and other studies | 31 | 32.98 | 23.47, 42.48 |
| **22.** Interpretation consistent with pilot trial objectives and findings, balancing potential benefits and harms, and considering other relevant evidence | 11 | 91.67 | 86.08, 97.25 |
| **22a.** Implications for progression from pilot to future definitive trial, including any proposed amendments | 18 | 19.15 | 11.19, 27.10 |
| **23.** Registration number for pilot trial and name of trial registry | 24 | 25.53 | 16.72, 34.35 |
| **24.** Where the pilot trial protocol can be accessed, if available | 7 | 77.78 | 69.37, 86.18 |
| **25.** Sources of funding and other support (such as supply of drugs), role of funders | 72 | 76.60 | 68.04, 85.15 |
| **26.** Ethical approval or approval by research review committee, confirmed with reference number | 34 | 36.96 | 27.20, 46.71 |

**Supplemental Table 3.** Univariable Analysis of Additional Factors Associated with Completeness of Reporting

According to the CONSORT 2010 Extension for Pilot/Feasibility Trials
